# Successful treatment of pulmonary mucormycosis caused by *Rhizopus microsporus* with posaconazole

**DOI:** 10.1186/s40001-021-00602-x

**Published:** 2021-11-14

**Authors:** F. Yuan, J. Chen, F. Liu, Y. C. Dang, Q. T. Kong, H. Sang

**Affiliations:** 1 Sch Med, Southeast Univ, Nanjing, 210009 People’s Republic of China; 2 Dept Dermatology, Sch Med, Jinling Hosp, Nanjing Univ, Nanjing, 210002 People’s Republic of China

**Keywords:** Pulmonary mucormycosis, *Rhizopus microspores*, Diabetes mellitus, Posaconazole

## Abstract

**Background:**

Mucormycosis is a rare fungal infection occurring chiefly in the lung or the rhino-orbital-cerebral compartment, particularly in patients with immunodeficiency or diabetes mellitus. Among *Mucorales* fungi, *Rhizopus* spp. are the most common cause of mucormycosis.

**Case presentation:**

We report a case of pulmonary mucormycosis caused by *Rhizopus microsporus* in a young patient with diabetes but no other apparent risk factors. The diagnosis mainly relied on clinical manifestation, positive pulmonary tissue biopsy, and fungal culture. The patient was successfully treated with posaconazole oral suspension and remains asymptomatic at one-year follow-up.

**Conclusions:**

Pulmonary mucormycosis is a life-threatening condition and posaconazole is an effective treatment for pulmonary mucormycosis caused by *Rhizopus microspores*.

## Background

Mucormycosis is a rare opportunistic infection, but is associated with high mortality and morbidity. Mucormycosis occurs most commonly in immunocompromised patients, such as those with poorly controlled diabetes mellitus, solid organ or hematopoietic stem cell transplantation, trauma, and those receiving immunosuppressive therapy [1–3]. The most common site of infection was rhino-orbital-cerebral, followed by pulmonary. Pulmonary mucormycosis (PM) is a life-threatening condition if there is delay in diagnosis and treatment. A retrospective review found that, in zygomycosis, the mortality for diabetes was 44%, whereas PM mortality increased to 76% [4]. Here, we present a case of PM caused by *Rhizopus microsporus* in a patient with diabetes mellitus.

## Case presentation

A 26-year-old male carpenter presented to our hospital with low-grade fever, cough, expectoration, and stethalgia for one month. Several days later, his symptoms were aggravating then he was initially admitted to a local hospital where he was diagnosed with pneumonia and empirically treated with cephalosporins. On laboratory investigations, total white blood cell count (WBC) was 17.19 × 10^9^/L with 74.1% neutrophils. His temperature fluctuated between 37.0 and 38.4 ℃. Serial chest X-rays showed pneumonia in the right upper and lower lobe of lung and left lower lobe of lung. Computed tomography (CT) of the chest demonstrated inflammation of both lungs, but the patient lost the exact report.

**Fig1:**
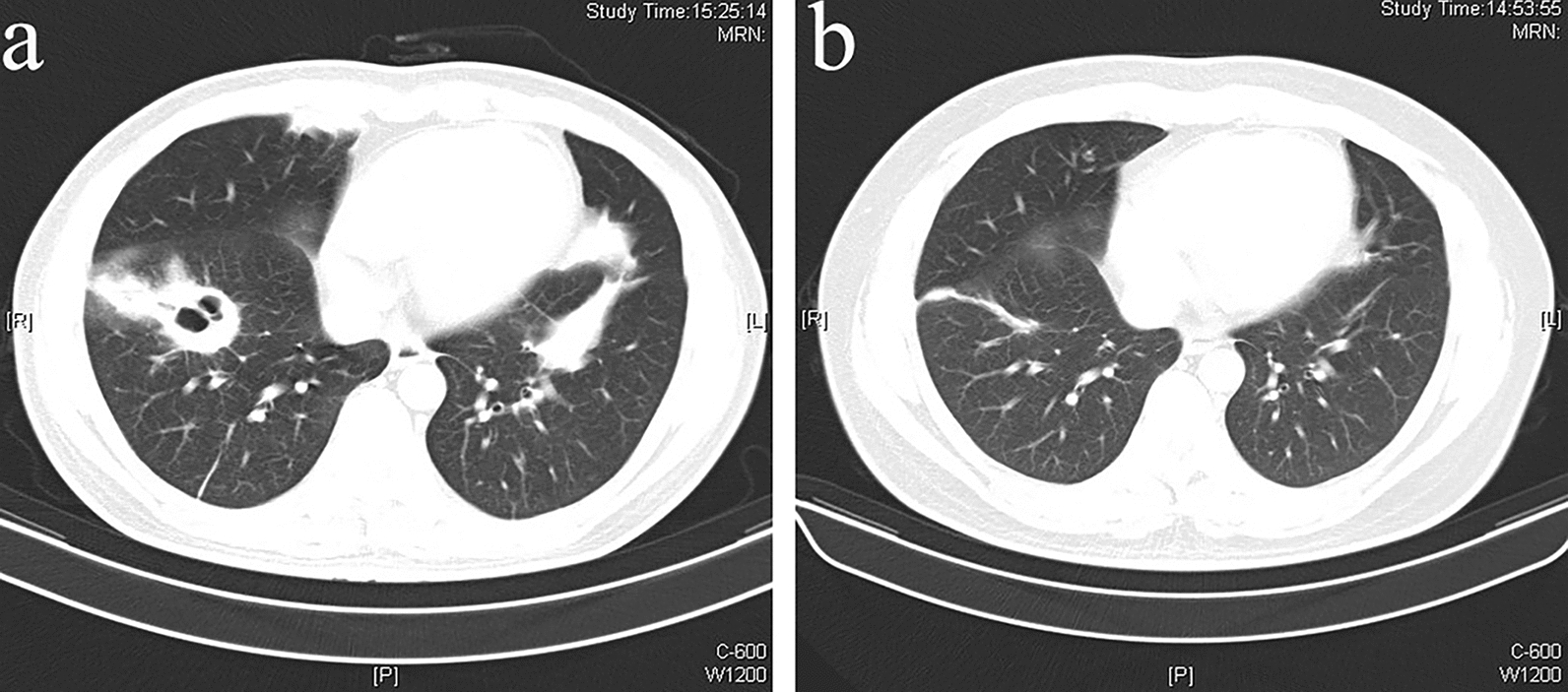
**a** Axial image from CT of the chest demonstrating bilateral patchy consolidation with pulmonary cavitation formation. **b** A follow-up CT scan after 6 months of antifungal treatment indicated the lesions nearly resolved

Four days later, antibiotic therapy was changed to vancomycin and sulperazone due to sputum culture reported the growth of *Gram-positive cocci*. A week after the treatment changed, a reexamination of thoracic CT showed patchy consolidation in both lungs with pulmonary cavitation formation (Fig. 1a). Therefore, he came to our hospital for further treatment. The patient had a 2-year history of type 2 diabetes and no history of hypertension, coronary heart disease, tuberculosis, chronic obstructive pulmonary disease, or chronic bronchitis. He did not take oral anti-diabetic drugs regularly and did not monitor blood glucose.

**Fig2:**
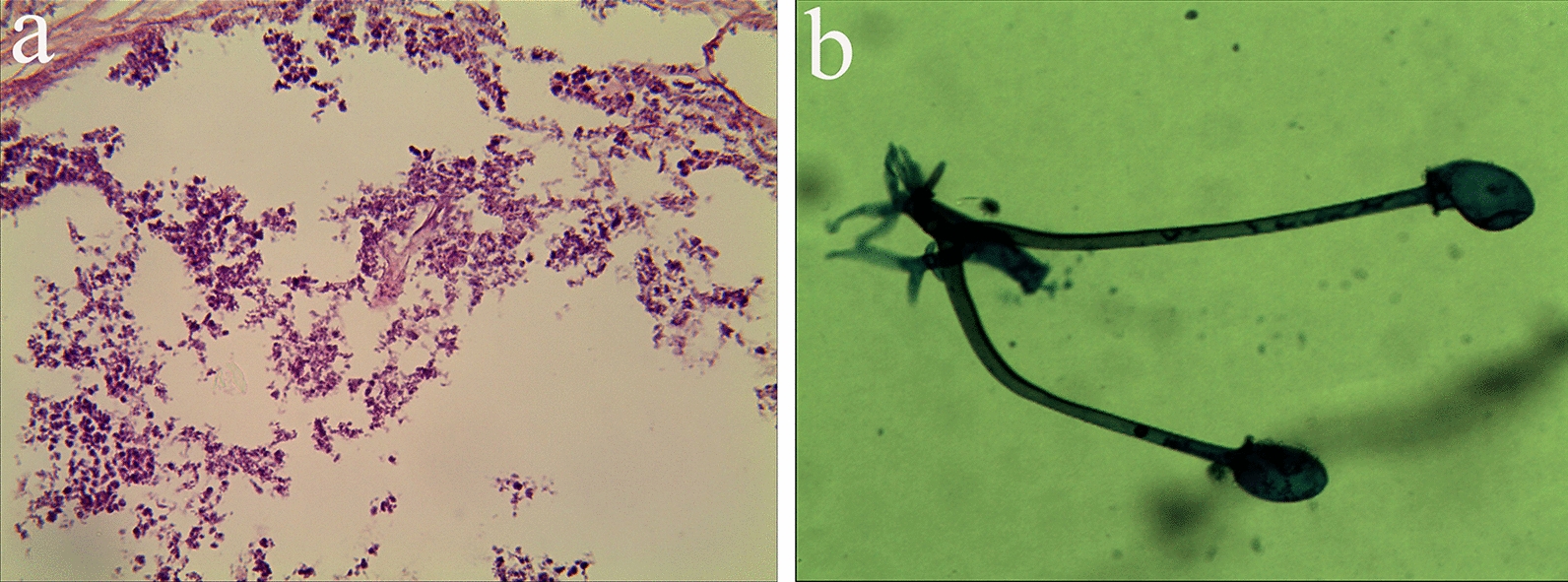
**a** Periodic acid–Schiff (PAS) stain presented broad, thin-walled, ribbon-like, aseptate fungal hyphae and right-angled hyphal branches. And the tissue was infiltrated by numerous lymphocytes (PAS × 400). **b** Light microscope examination showed that brownish sporangiophores were in pairs branching from nodal rhizoids (× 400)

His glycated hemoglobin (HbA1c) was 12.3%, creatinine and blood urea nitrogen were normal. Furthermore, the third thoracic CT shows multiple cavitary lesions in both lungs. Considering the patient’s CT images show a trend of gradual progress, we gave him a bronchoscopy. Bronchoscopy revealed sticky purulent secretions in the left and right main bronchus, in each segment bronchus, especially in the left upper anterior lobe, the left lingual lobe, the left medial basal segment of the lower lobe, and the left dorsal segment of the lower lobe. Bacterial culture showed no growth of pathogenic bacteria. The outcome of Ziehl–Neelsen staining was negative. Histopathology showed the disappearance of the alveolar structure, fibroplasia, and necrosis. Periodic acid–Schiff (PAS) staining of the tissue presented broad, thin-walled, and aseptate fungal hyphae as well as right-angled hyphal branches (Fig. 2a). The samples were inoculated on Sabouraud dextrose agar (SDA) media under 30 ℃ and white colonies appeared after 5 days. The fungus was then subcultured on potato dextrose agar (PDA) plates. Light microscope examination showed that nodal rhizoids branched a pair of brownish sporangiophores and sporangiospores were hyaline, angular, and broadly ellipsoidal (Fig. 2b).

For further identification, the ITS regions were sequenced with primer ITS1 and primer ITS4. The sequence of the isolate aligned with 99% similarity to multiple sequences (e.g., Accession Number KM103772.1) of *Rhizopus microsporus* available in GenBank database. Temperature tests revealed that the isolate grew well at 37 ℃, 40 ℃ and 45 ℃, but did not grow at 50 ℃. Antifungal susceptibility was performed in accordance with the CLSI M38-A guidelines [5].

The samples were inoculated on SDA media. The isolate was sensitive to posaconazole and amphotericin B, with resistance to fluconazole and itraconazole. Taking the drug antifungal susceptibility into consideration, the patient was supervised to the following regimen: posaconazole oral suspension (400 mg twice a day, along with a high-fat diet), ambroxol (30 mg, three times a day), repaglinide (1 mg, three times a day) combined with low-sugar, high-fiber diets. After approximately 6 months of treatment with posaconazole oral suspension, the patient was well and asymptomatic, and the lesions completely resolved (Fig. 1b).

## Discussion and conclusion

PM is an uncommon presentation in diabetic patients, but is associated with high mortality and morbidity. An early diagnosis of PM is difficult, due to the rarity of the disease and clinical and radiological features resembling tuberculosis (TB).

Upon suspicion of mucormycosis, European Confederation of Medical Mycology (ECMM) strongly recommended appropriate imaging to document the extent of disease and an early complete surgical treatment whenever possible, in addition to systemic antifungal therapy [6]. First-line treatment with high-dose liposomal amphotericin B is strongly recommended, followed by intravenous isavuconazole and intravenous or delayed-release tablet posaconazole. A study of patients with hematological diseases showed that posaconazole oral suspension is effective as a salvage therapy for invasive fungal infections [7]. Both triazoles are strongly recommended in salvage treatments. Amphotericin B deoxycholate is not recommended, because of substantial toxicity, except the only option in resource-limited conditions. Due to nonspecific clinical presentation, a clinician must keep a high index of suspicion in mind especially in diabetic patients, and pursue invasive testing early to establish a prompt diagnosis. ECMM clinical guidelines strongly recommend direct microscopy, histopathology, and culture for the diagnosis [6].

The current case is a 26-year-old uncontrolled diabetic patient whose work as a carpenter exposes him to a wide range of fungal spores. A study showed that airborne spores of *Rhizopus microsporus* were frequently found in filter samples of wood trimmers [8]. Consequently, work-related direct inhalation of the *Rhizopus microsporus* was most probably the cause of the pulmonary infection. A retrospective cohort study showed that the incidence of breakthrough invasive fungal infections was not significantly different between patients receiving the tablet formulation and those receiving the oral suspension formulation among patients with hematologic malignancies [9]. After comprehensive consideration, our patient was treated with posaconazole oral suspension and repaglinide. Surgical opinion was sought, but was not advisable due to bilateral lung involvement. After approximately 6 months of treatment with posaconazole oral suspension, the patient was well and asymptomatic, and the lesions improved considerably on thoracic CT.

In conclusion, we have described that posaconazole successfully cured a 26-year-old uncontrolled diabetic patient suffering from pulmonary infection caused by *Rhizopus microsporus*. The observations in our case emphasize the importance of considering fungal infections in patients with common diseases, such as untreated diabetes mellitus, and with long-term occupational exposure history.

## Data Availability

Not applicable.

## Abbreviations

PM: Pulmonary mucormycosis
WBC: Blood cell count
CT: Computed tomography
PAS: Periodic acid–Schiff
SDA: Sabouraud dextrose agar
PDA: Potato dextrose agar
TB: Tuberculosis
ECMM: European Confederation of Medical Mycology
